# Deletion of P58^IPK^, the Cellular Inhibitor of the Protein Kinases PKR and PERK, Causes Bone Changes and Joint Degeneration in Mice

**DOI:** 10.3389/fendo.2014.00174

**Published:** 2014-10-17

**Authors:** Sophie J. Gilbert, Lee B. Meakin, Cleo S. Bonnet, Mari A. Nowell, Warren C. Ladiges, John Morton, Victor C. Duance, Deborah J. Mason

**Affiliations:** ^1^Pathophysiology and Repair Division, Arthritis Research UK Biomechanics and Bioengineering Centre, School of Biosciences, Cardiff University, Cardiff, UK; ^2^School of Veterinary Sciences, University of Bristol, Bristol, UK; ^3^Section of Inflammation, Skin and Joint Disease, Institute of Infection and Immunity, School of Medicine, Cardiff University, Cardiff, UK; ^4^Department of Comparative Medicine, School of Medicine, University of Washington, Seattle, WA, USA

**Keywords:** P58^IPK^, bone, articular cartilage, osteoarthritis, PERK, PKR

## Abstract

**Objective:** Protein kinase-like endoplasmic reticulum kinase (PERK) and protein kinase R (PKR) are implicated in endoplasmic reticulum stress-induced arthritis and pro-inflammatory cytokine-mediated cartilage degradation *in vitro*, respectively. We determined whether knockout of the cellular inhibitor of PERK and PKR, P58^IPK^ causes joint degeneration *in vivo* and whether these molecules are activated in human osteoarthritis (OA).

**Materials and Methods:** Sections of knee joints from P58^IPK^-null and wild-type mice aged 12–13 and 23–25 months were stained with toluidine blue and scored for degeneration using the osteoarthritis research society international (OARSI) system. Bone changes were assessed by radiology and high-resolution micro-computed tomography of hind limbs. Sections from the medial tibial plateaus of two human knees, removed in total knee replacement surgery for OA, were immunolabelled for phosphorylated PERK and PKR and P58^IPK^.

**Results:** Knockout mice exhibited narrower tibiae (*p* = 0.0031) and smaller epiphyses in tibiae (*p* = 0.0004) and femora (*p* = 0.0214). Older knockout mice had reduced total volume inside the femoral periosteal envelope (*p* = 0.023), reduced tibial (*p* = 0.03), and femoral (*p* = 0.0012) bone volumes (BV) and reduced femoral BV fraction (*p* = 0.025). Compared with wild-types, younger P58^IPK^-null mice had increased OARSI scores in medial femoral condyles (*p* = 0.035). Thirty four percent of null mice displayed severe joint degeneration with complete articular cartilage loss from the medial compartment and heterotopic chondro-osseous tissue in the medial joint capsule. Phosphorylated PERK and PKR were localized throughout human osteoarthritic tibial plateaus but, in particular, in areas exhibiting the most degeneration. There was limited expression of P58^IPK^.

**Conclusion:** This study is the first to reveal a critical role for P58^IPK^ in maintaining joint integrity *in vivo*, implicating the PKR and PERK stress signaling pathways in bony changes underlying the pathogenesis of joint degeneration.

## Introduction

Osteoarthritis (OA) is a disease of the synovial joint associated with pathological changes in the bone and cartilage that cause pain and disability. Abnormal mechanical load and obesity represent primary risk factors for OA development and progression (1, 2). Several studies have implicated the stress-activated protein kinase R (PKR) and protein kinase-like endoplasmic reticulum kinase (PERK) signaling pathways in the pathogenesis of arthritic disease (3–9). PKR is constitutively expressed in all cells, with levels increasing upon cellular stress [reviewed in Ref. (10)]. In chondrocytes, constitutive PKR expression is known to be high (8), with further activation (phosphorylation) resulting from cellular stress leading to up-regulation of matrix degrading enzymes (3, 8), proteoglycan degradation (3, 4), phosphorylation of eukaryotic initiation factor 2 alpha (eIF2α) (11) and down-regulation of protein synthesis [reviewed in Ref. (10)]. Furthermore, the PKR pathway is mechano-responsive, gene expression being up-regulated (12) at the onset of load-induced OA (13) and mediating translational arrest induced by cyclic mechanical load in articular cartilage (14). The cellular level of PKR increases as tissues age, concomitant with a decrease in the rate of protein synthesis (15), implicating PKR in the pathogenesis of age-related diseases such as OA. PERK (16) and PKR (17, 18) are also phosphorylated during endoplasmic reticulum (ER) stress, leading to eIF2α phosphorylation and down-regulation of protein synthesis, thus, reducing the load of protein passing through the ER. Cells that have a high-secretory capacity, including pancreatic β cells, chondrocytes, and osteoblasts, are hyper-sensitive to ER stress induction (19–21).

P58^IPK^ (58 kDa inhibitor protein kinase), is a member of the heat shock protein 40 family and has several known interacting partners allowing it to have diverse functions in the cell; it is localized to the cytosol where it acts as a cellular inhibitor of PKR and PERK activity and thus regulator of global protein synthesis and translocates to the ER during ER stress whereby it acts as a chaperone in the ER lumen. It is ubiquitously expressed, abundant in secretory cells of the pancreas and liver (15, 22), shows increased expression with age (15) and plays important roles in maintaining cellular homeostasis (23–27). In its protective role as a molecular chaperone, it interacts with unfolded proteins such as luciferase, rhodanese, and insulin to prevent their aggregation (28) and binds BiP to facilitate misfolded protein folding (29, 30). In addition, it has also been shown to bind to the ER protein translocation channel Sec61 to play a role in ER protein degradation (31). P58^IPK^ mRNA and protein levels are increased in response to ER stress to inhibit PERK and PKR activity and restore homeostasis (23, 24, 32). Knockdown of P58^IPK^ using siRNA in HEK-293 and HeLa cells increased levels of total PKR and phosphorylated PERK, with subsequent eIF2α phosphorylation, inhibition of protein synthesis and apoptosis (24). Knockdown of P58^IPK^ increased release of tumor necrosis factor-alpha (TNF-α) in rat retinal cells (27), phosphorylation of eIF2α and expression of ER stress response proteins in embryonic stem cells (23). Furthermore, P58^IPK^-null cells have heightened basal levels of ER stress (26). P58^IPK^-null mice are smaller with lower body weights than heterozygous and wild-type mice, reduced body fat, hypoinsulinemia, and gradual onset diabetes (25, 26). However, up until now, skeletal phenotype has not been assessed in P58^IPK^-null mice.

Given the role of PERK and PKR in cell signaling events associated with arthritis, we tested the hypothesis that absence of P58^IPK^ results in a degenerative joint phenotype *in vivo*. The aims of this observational study were to describe a phenotypic characterization of donated knee joints from a P58^IPK^ knockout mouse colony.

## Materials and Methods

### Materials

All chemicals were obtained from Sigma (Poole, UK) unless otherwise stated and were of analytical grade.

### Tissue

#### Human OA

The medial tibial plateau (MTP) was obtained from two randomly selected patients undergoing total knee replacement for OA (female, 83 and 59 years old). Informed consent was gained from each donor according to protocols approved by the Research Ethics Committee for Wales (Ref: 10/MRE0928; Cardiff & Vale Heath Board R&D approval Ref: 10/OAE/497).

#### P58^IPK^-null mice

Hind limbs were obtained from previously generated male P58^IPK^-null and age-matched wild-type mouse colonies as described (25) and approved by the University of Washington Institutional Animal Care and Use Committee. Two age groups were analyzed: 12–13 and 23–25 months. Mice were 100% C57Bl/6 and had been maintained on a C57Bl/6 background by alternate generation backcrossing. All comparisons are between null and wild-types of 12–13 months (four null, and three wild-type) and 23–25 months mice (five null, four wild-type). Where analyses deviate from this standard comparison, the numbers of animals used are clearly stated in the figure legends.

### Specimen preparation

#### Human material

Tissue was fixed (2 days, 10% neutral buffered formalin), decalcified (4°C, 10% EDTA, Fisher Scientific), paraffin embedded, and coronally sectioned (6 μm).

#### Mouse material

Hind limbs were immediately fixed post-mortem in formalin. For histology and immunohistochemistry, right knee joints were decalcified as described in Section “Human Material.” Joints were fixed at 90° and embedded frontally in paraffin blocks for coronal sectioning parallel to the tibia. Serial sections (5 μm) obtained at 100 μm intervals through the joint, were dewaxed and rehydrated prior to staining with toluidine blue or processing for immunohistochemistry. Left knee joints were stored in 70% ethanol prior to radiological and high-resolution micro-computed tomography (μCT) analysis.

### Histological scoring

#### Human MTP

The human MTPs had been previously stained with toluidine blue (33) and scored for degenerative changes. Sections were shown to have cartilage fibrillation, proteoglycan loss, and breach of the tidemark. Cartilage degradation severity (indicated by increasing Mankin score) increased toward the outer edge of the MTP (33).

#### Mouse knees

The medial femoral condyle (MFC), MTP, lateral femoral condyle (LFC), and lateral tibial plateau (LTP) from two sections (either side of the center of the joint, approximately 200 μm apart) were scored for degenerative changes by two independent observers blinded to genotype. For each mouse, a single score representing the mean value from both observers and sections was used for statistical comparisons. The osteoarthritis research society international (OARSI) scoring method (34) was used with toluidine blue staining utilized instead of safranin O. The OARSI system uses a 0–6 subjective score for osteoarthritic changes (Table S1 in Supplementary Material: parameter 1), 0–3 for subchondral bone changes (Table S1 in Supplementary Material: parameter 2), and 0–5 for proteoglycan depletion (Table S1 in Supplementary Material: parameter 3). This gives a total score of 14 for each quadrant (parameters 1–3).

### Radiological analysis

A KODAK *In vivo* Imaging System FX Pro (AMV, Lincoln, UK) was used to produce high-resolution digital radiographs of medial and frontal views of the left mouse hind legs. Capture settings were as follows: 2× binning, 0.4 mm aluminum filter, 30 s exposure, KVP 35, F-stop 3.99, FOV 22.2 mm. A region of interest (ROI), of the same size and shape, and located as near to the inferior tibio-fibular joint as possible, was defined (see Figure S1 in Supplementary Material). Placing the ROI as close to this joint as possible provided us with a landmark allowing us to make direct comparisons of the same region across animals. The exact location was defined from the Chi-square value, derived from the bone density analysis, which is a measure of the fitting quality in the non-linear least-squares fit of the cylinder to the bone segment. The ROI was analyzed for changes in long-bone density (g/cm^3^) using Carestream Molecular Imaging Software (version 5.3.3) with bone density software module (35). In addition, a ROI was drawn around any calcification within the Achilles to measure its area (pixels) and a linear ROI used to measure the height and width of the tibia at the tibio-fibular joint (millimeter) (Figure S1 in Supplementary Material).

### High-resolution μCT analysis

Left mouse knees were imaged using the SkyScan 1172 (Bruker, Kontich, Belgium) with a voxel size of 4.8 μm (110 mm^3^) using previously reported scanning and reconstruction methods (36). The ROI incorporated 25 lines (0.25 mm) starting from the central line of the epiphyses and moving proximally for the femur and distally for the tibia. We evaluated the effect of genotype and age on epiphyseal total volume inside the periosteal envelope (TV), bone volume (BV), medullary volume (MV), and bone volume fraction (BV/TV) according to ASBMR guidelines (37). Femoral and tibial epiphyseal height, excluding the growth plate, is also reported (Figure S1 in Supplementary Material) and 3D reconstructions generated.

### Immunohistochemistry

Active PERK and PKR and their endogenous inhibitor were immunolocalized in sequential sections from two human MTP using antibodies to phosphorylated PERK (Santa Cruz: sc-32577; 1:50), phosphorylated PKR (Santa Cruz: sc-101783; 1:50), and P58^IPK^ (Abcam: ac70840; 1:20). Sections were deparaffinized and rehydrated prior to antigen retrieval (1 mg/mL trypsin for 1 h at 37°C). Each subsequent step was performed at room temperature unless stated otherwise and between each incubation step, sections were washed 3 min × 5 min in 0.01 M phosphate buffered saline (PBS, pH 7.4) containing 0.001% (v/v) Tween 20 (wash buffer). All antibodies were diluted in wash buffer. Endogenous peroxidase activity was blocked with 0.3% (v/v) hydrogen peroxide for 30 min. Sections were subsequently treated with 10% normal goat serum for 1 h prior to overnight incubation (4°C) with primary antibody. Biotinylated secondary antibody was applied and incubated for 30 min detection (Vectastain Elite ABC kit, nickel enhanced diaminobenzidine, Vector Laboratories). Sections were finally dehydrated, cleared in xylene, and mounted. Slides were viewed on a Leica DMRB microscope. IgG controls were negative (Figure S2 in Supplementary Material).

### Data analysis

For all statistics, unless stated otherwise, hindlimb characteristics were compared between null and wild-type mice of 12–13 months (four null, and three wild-type) and 23–25 months mice (five null, four wild-type). To assess and compare sample distributions, graphs show box and whisker plots of minimum and maximum values, 25th and 75th quartiles and median; outliers revealed by these plots were removed prior to further analysis. Data were tested for normality and equal variances prior to analysis (Minitab 16). Data were analyzed by general linear model analysis of variance (GLM ANOVA) with two factors (age and genotype) followed by Tukey’s *post hoc* test. Differences were considered significant at *p* ≤ 0.05.

## Results

### P58^IPK^-null mice have narrower tibias and shorter epiphyses

**Figure 1:**
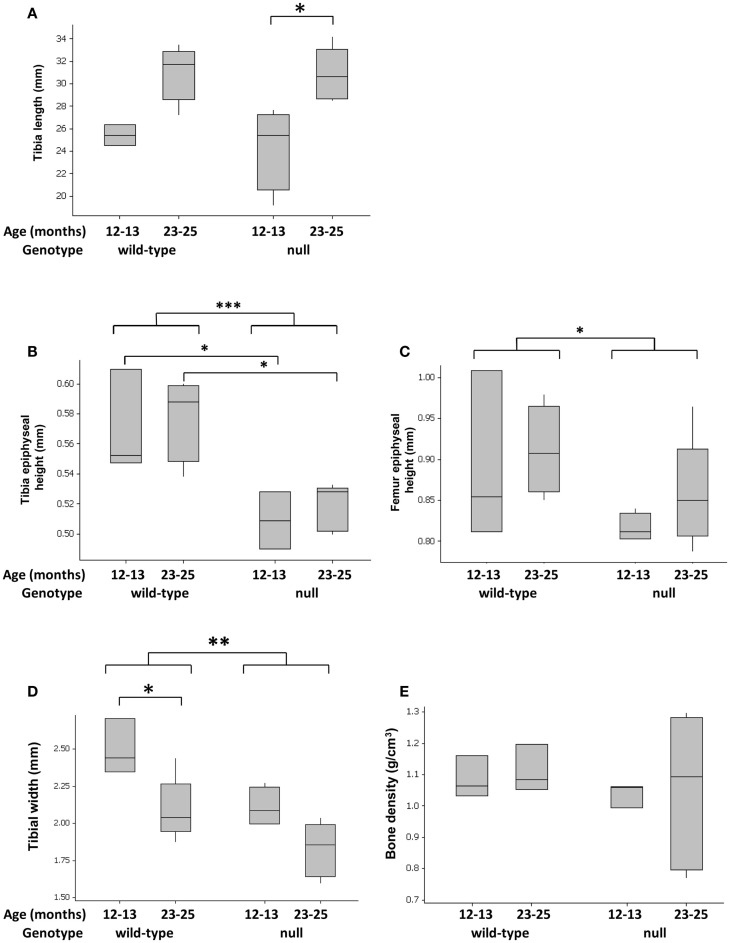
**Tibial widths and epiphyseal heights are reduced in P58^IPK^-null mice**. Radiographs and high-resolution μCT of the left limb from wild-type and null mice were used to measure tibial lengths **(A)**, tibial epiphyseal heights **(B)**, femoral lengths **(C)**, and tibiae widths at the tibia/fibula intersect **(D)**. Tibial bone density was measured close to the inferior tibio-fibular joint of wild-type and null mice **(E)**, [Figure S1 in Supplementary Material]. Significant differences were detected by GLM ANOVA and Tukey’s *post hoc* test: *p ≤ 0.05; **p < 0.01; ***p < 0.001. For tibial length, width, and bone density measurements: 12–13-month-old mice *n* = 4 null, *n* = 3 wild-type and 23–25-month-old mice *n* = 5 null, *n* = 4 wild-type. For micro CT measurements: 12–13-month-old mice *n* = 3 null, *n* = 3 wild-type and 23–25-month-old mice *n* = 5 null, *n* = 4 wild-type.

P58^IPK^-null mice exhibit a small body phenotype (Figure S3 in Supplementary Material) (25). Since no previous analysis of the long bones has been performed, we measured tibial length, width, and density, as well as height of the epiphyses. Loss of P58^IPK^ appeared not to affect the overall length of the tibia (Figure 1A; *p* = 0.886). However, P58^IPK^-null mice had significantly shorter tibial (*p* = 0.0004) and femoral epiphyses (*p* = 0.0214) compared to wild-type mice (Figures 1B,C). This difference occurred in the tibial epiphyses of both 12–13-month-old (*p* = 0.032) and 23–25-month (*p* = 0.016) old null mice compared to the age-matched wild-types. In addition, null mice exhibited a significant reduction in the width of their tibiae at the tibia/fibula intersect when compared to wild-type mice (*p* = 0.0031; Figure 1D). There appeared to be no difference in the densities of the long-bone situated close to the inferior tibio-fibular joint of wild-type and null mice (Figure 1E).

### Loss of P58^IPK^ causes joint degeneration

**Figure 2:**
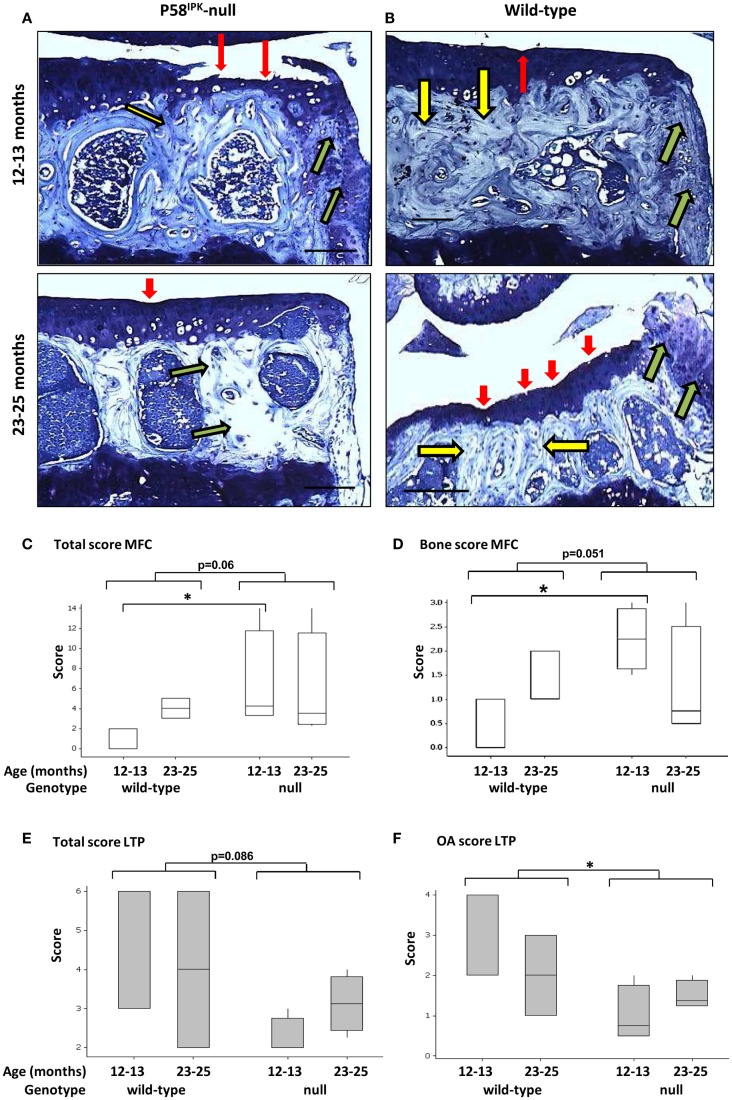
**Knee joint degeneration is increased in P58^IPK^-null mice**. Examples of toluidine blue stained coronal sections from the MTPs from P58^IPK^-null **(A)** and wild-type **(B)** mice from both age groups (12–13 months: top panels; 23–25 months: bottom panels) are shown representing typical signs of joint degeneration: cartilage tears and degeneration (red arrows); subchondral bone changes (yellow arrows); and osteophyte formation (green arrows). Scale bars = 500 μm. The OARSI scoring method (Table S1 in Supplementary Material) was used to determine whether null mice had degeneration over and above that commonly observed in C57Bl/6 wild-type mice. Total scores [parameters 1–3; **(C)**] and bone scores [parameter 2; **(D)**] from the MFC and total scores [parameters 1–3; **(E)**] and OA scores [parameter 1; **(F)**] from the LTP obtained using are shown. Significant differences were detected by GLM ANOVA and Tukey’s *post hoc* test: *p ≤ 0.05. LTP, lateral tibial plateau; MFC, medial femoral condyle. OARSI scores: 12–13-month-old mice *n* = 3 null, *n* = 3 wild-type and 23–25-month-old mice *n* = 4 null, *n* = 4 wild-type.

Joint degeneration was observed in both P58^IPK^-null (Figure 2A) and wild-type (Figure 2B) mice of both ages. These degenerative changes included cartilage tears (red arrows), subchondral bone changes (yellow arrows), and osteophyte formation (green arrows). To assess whether loss of P58^IPK^ resulted in joint degeneration over and above that expected in aging C57Bl/6 mice (38–41), knees were scored using an adaptation of the OARSI system (34). Despite the presence of degeneration in both groups, GLM ANOVA of OARSI scores revealed that 12–13-month-old null mice had fourfold higher total scores (sum of all three parameters; Table S1 in Supplementary Material) in the MFC compartment than their wild-type, age-matched controls (Figure 2C; *p* = 0.035 following rank transformation of data). Analysis of individual parameters revealed that there was a weak, but inconclusive increase in the MFC bone score in null mice (parameter 2) (Figure 2D; GLM ANOVA genotype *p* = 0.051 following log transformation of data) and that 12–13-month-old null mice had 6.7-fold higher bone scores (parameter 2) than age-matched wild-types (Figure 2D; *p* = 0.024 following log transformation of data). P58^IPK^-null mice had 1.5-fold lower total scores (sum of all three parameters; Table S1 in Supplementary Material) in the LTP compared to wild-type mice, but this difference was not significant (Figure 2E; GLM ANOVA genotype *p* = 0.086). However, OA scores (parameter 1) were significantly reduced in the LTP (Figure 2F; GLM ANOVA genotype *p* = 0.033 following log transformation of data).

**Figure 3:**
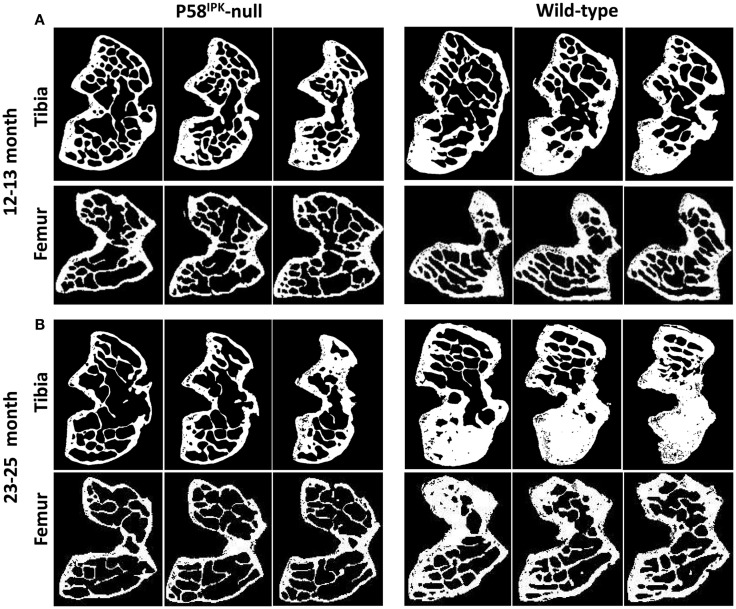
**Significant bone loss occurs in the knees of P58^IPK^-null mice**. MicroCT analysis of a ROI incorporating 25 lines (0.25 mm) starting from the central line of the epiphyses and moving proximally for the femur and distally for the tibia were obtained. Representative images are shown of slices taken from the top, middle and bottom of the 25 lines. **(A)** images from 12 to 13-month-old mice; **(B)** images from 23 to 25-month-old mice. In each slice, the lateral compartment is at the top and medial at the bottom.

High-resolution μCT analysis revealed a significant reduction in bone mass in P58^IPK^-null mice (Table 1). GLM ANOVA revealed a significant effect of genotype on tibial bone with loss of P58^IPK^ reducing TV (*p* = 0.002), BV (*p* < 0.0001), and BV/TV (*p* = 0.01). BV in 23–25-month-old null mice was significantly reduced compared to age-matched wild-types (*p* = 0.03). Individual μCT slices revealed that bone loss was largely confined to the medial plateau of the tibiae (Figure 3).

**Table 1:** **Bone parameters measured using high-resolution μCT**.

	P58^IPK^-null		Wild-type	
	Tibia	Femur	Tibia	Femur
TV (mm^3^)
12–13 months	$\begin{array}{c} 0.404\pm 0.014\\ 0.397\pm 0.013 \end{array}\}**$	$\begin{array}{c} 0.416\pm 0.010\\ 0.394\pm 0.014^{\text{a}} \end{array}\}*$	0.472 ± 0.024	0.437 ± 0.031
23–25 months			0.450 ± 0.013	0.484 ± 0.022
BV (mm^3^)
12–13 months	$\begin{array}{c} 0.231\pm 0.012\\ 0.175\pm 0.009^{\text{a}} \end{array}\}***$	$\begin{array}{c} 0.194\pm 0.008\\ 0.150\pm 0.008^{\text{b}} \end{array}\}***$	0.276 ± 0.007	0.224 ± 0.019
23–25 months			0.248 ± 0.029	0.239 ± 0.016
BV/TV (%)
12–13 months	$\begin{array}{c} 57.37\pm 2.84\\ 44.17\pm 2.21 \end{array}\}*$	$\begin{array}{c} 46.70\pm 2.75\\ 37.95\pm 1.39^{\text{a}} \end{array}\}**$	58.59 ± 2.21	51.10 ± 1.51
23–25 months			55.52 ± 7.12	49.47 ± 3.87
MV (mm^3^)
12–13 months	0.172 ± 0.013	0.222 ± 0.017	0.197 ± 0.020	0.213 ± 0.014
23–25 months	0.222 ± 0.013	0.244 ± 0.010	0.201 ± 0.034	0.245 ± 0.025

*Bone parameters obtained from μCT analysis of a ROI incorporating 25 lines (0.25 mm) starting from the central line of the epiphyses and moving proximally for the femur and distally for the tibia*.

*TV, total volume within the periosteal envelope; BV, bone volume; BV/TV, bone volume fraction; MV, marrow volume*.

Data represented as mean ± SEM (*n* = 3–5). Significant differences were detected by GLM ANOVA and Tukey’s *post hoc* test. Genotype effects are shown *p < 0.05; **p < 0.01; ***p < 0.001 compared to the same compartment in wild-type mice.

*Age effects are shown ^a^*p* < 0.05; ^b^*p* < 0.01 compared to the same compartment in age-matched wild-type mice*.

General linear model analysis of variance also revealed a significant effect of genotype on the femoral bone with loss of P58^IPK^ reducing TV (*p* = 0.013), BV (*p* = 0.0005), and BV/TV (*p* = 0.007). Femora from 23 to 25-month-old mice were smaller than those from age-matched wild-type mice with 18% reduction in TV (bone volume + marrow volume; *p* = 0.023), 37% reduction in BV (*p* = 0.0012), and a significant overall reduction of femoral BV fraction (BV/TV; *p* = 0.025). Individual slices reveal that bone loss occurred across the femoral condyle (Figure 3).

### A severe degenerative phenotype occurs in a subset of P58^IPK^-null mice

**Figure 4:**
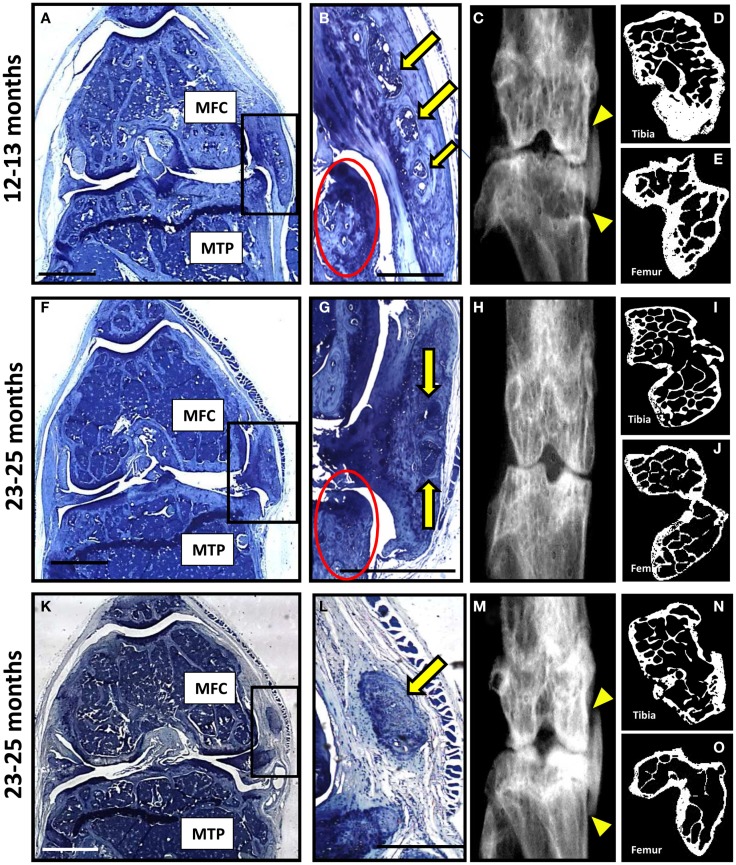
**A subset of P58^IPK^-null mice reveal a severe degenerative joint phenotype**. Coronal sections, stained with toluidine blue, are depicted from the right knee joints of three of the P58^IPK^-null mice aged 12–13 months **(A)** and 23–25 months **(F,K)** old with the severe phenotype. Significant bone remodeling and cartilage loss can be seen in the MTP and MFC. Magnified views of the heterotopic chondro-osseous tissue observed in the medial capsule of these sections (box) are depicted **(B,G,L)** showing areas of bone marrow formation (yellow arrows) along with osteophyte formation (red circles) in two of the mice. Coronal radiographs from the contra-lateral legs of each animal depicted in **(A,F,K)** are shown **(C,H,M)** highlighting the ectopic radio-dense areas in the medial collateral ligaments and the joint capsule of two of these mice [**(C,M)**; yellow triangles]. High-resolution μCT images of the contra-lateral leg revealed changes in joint shape and osteophyte formation on the tibiae **(D,I,N)** and femurs **(E,J,O)**. Scale bar = 500 μm except for **(A,F,K)** = 1 mm.

A very severe degenerative phenotype, not seen in any of the seven wild-type mice, was observed in a third of null mice; one from the 12–13 month group (Figures 4A–E) and two from the 23–25 month group (Figures 4F–O). The degeneration included loss of cartilage with erosion through to the subchondral bone, abnormal bone changes, and large osteophytes within the medial side of the joint. Moderate cartilage erosion was also observed in the lateral joint compartment of these mice. Heterotopic chondro-osseous tissue formed within the medial joint of each of the three mice with severe degeneration (Figures 4B,G,L), which was not observed in any other mice. Ectopic bone within the medial collateral ligaments and the joint capsule was observed within the contra-lateral leg of two of these mice (Figures 4C,M). High-resolution μCT images revealed changes in joint shape and osteophyte formation on the tibiae (Figures 4D,I,N) and femurs (Figures 4E,J,O) of these mice with severe degeneration. The extensive ectopic bone formation was further evident in frontal and side view 3D reconstructions from the μCT scans (Figure S4 in Supplementary Material). In addition to the two age groups included in this study, we also examined four 18-month-old mice (wild-type *n* = 2; null *n* = 2) and found that this severe phenotype was observed in one of the null mice (Figure S5 in Supplementary Material).

### P58^IPK^-null mice have less calcification in their Achilles tendon

**Figure 5:**
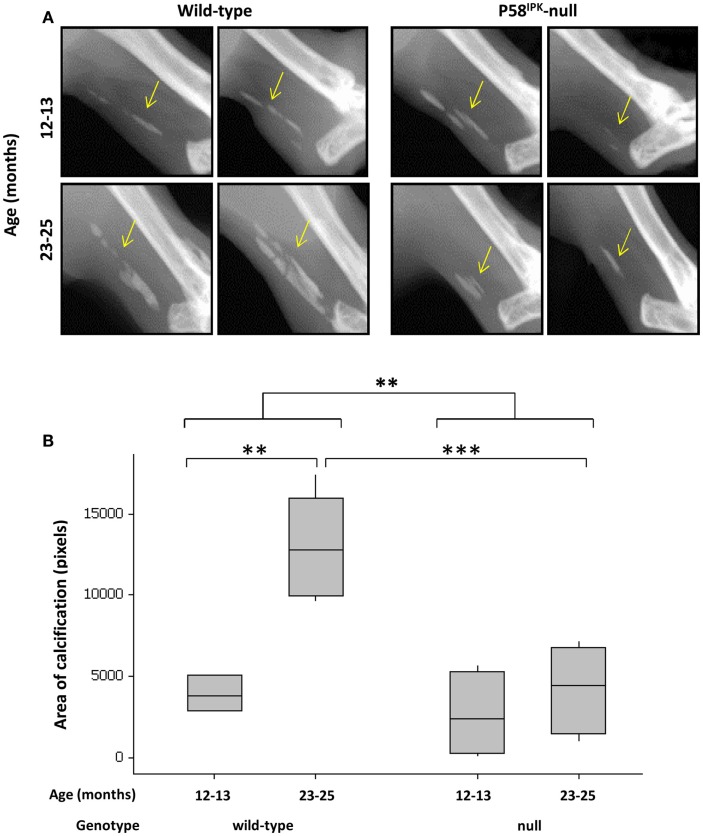
**Achilles tendon calcification is reduced in P58^IPK^-null mice**. **(A)** Radiographs from the left leg of wild-type and null mice show areas of calcification within the Achillles tendon (yellow arrow). A ROI was drawn around the calcified tissue and bone density software used to measure the area of calcification **(B)** with significant differences detected by GLM ANOVA and Tukey’s *post hoc* test: **p < 0.01; ***p < 0.001. Achilles calcification area: 12–13-month-old mice *n* = 5 null, *n* = 3 wild-type and 23–25-month-old mice *n* = 5 null, *n* = 4 wild-type.

Achilles tendon calcification occurred in 95% of all mice (Figure 5A), with P58^IPK^-null mice exhibiting significantly less calcification than wild-types (*p* = 0.002, Figure 5B). There was an age-related increase in Achilles calcification in wild-type mice (3.3-fold increase in 23–25-month-old mice compared to 12–13-month-old mice, *p* = 0.0023). Null mice showed a threefold reduction in calcified tissue at 23–25 months compared to age-matched wild-types (*p* = 0.0009).

### Expression levels of phosphorylated PKR and PERK in human OA joint tissues correlate to the severity of degeneration

**Figure 6:**
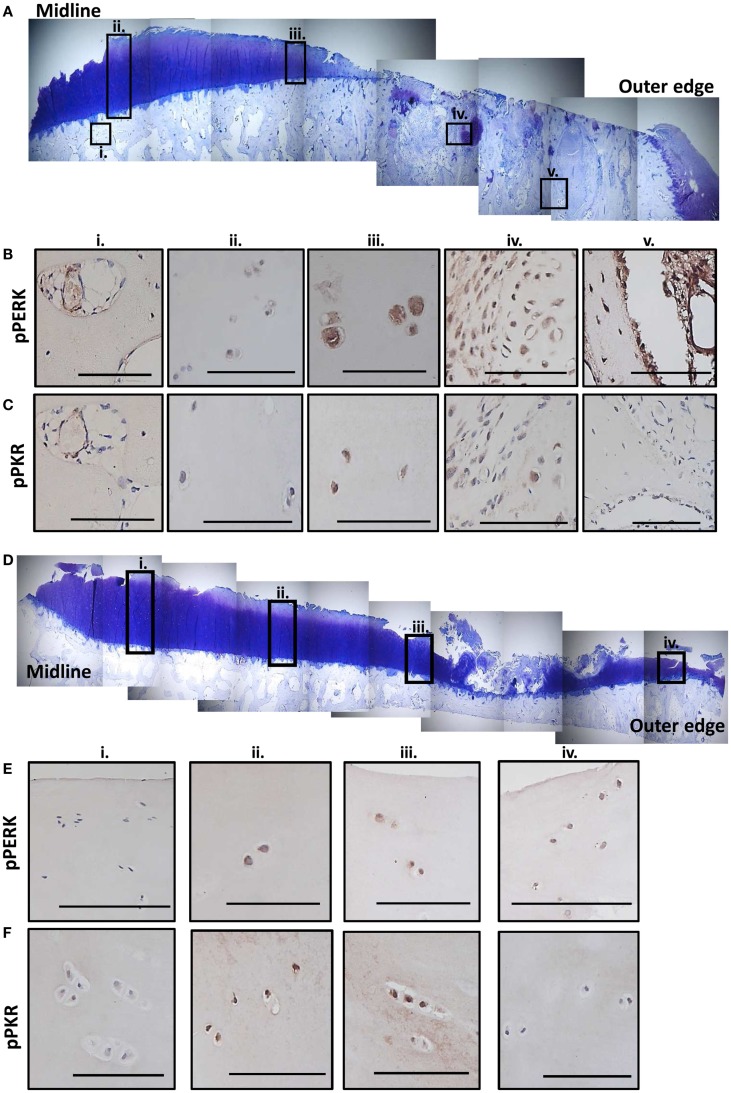
**Expression of phosphorylated PKR and PERK correlate with the degree of degeneration in human OA tissue**. Sections from the MTP of two patients undergoing total knee replacement for OA were stained with toluidine blue to establish the degree of tissue degeneration. The subcellular localization of phosphorylated PKR and PERK were determined by immunohistochemistry. Patient 1 had complete cartilage loss and severe bone changes toward the outer edge of the plateau with the remaining cartilage showing signs of significant fibrillation and proteoglycan loss **(A)**. Five regions (black boxes; i–v) highlight areas of limited damage (joint middle) to severe damage (outer edge). Representative staining for phosphorylated PERK **(B)** and PKR **(C)** from these five regions are shown revealing more staining in the cartilage as the damage progresses outwards and significant activation in the remodeling bone in the areas underlying the complete loss of cartilage. Patient 2 had cartilage fibrillations and proteoglycan loss, which were more extensive toward the outer edge but the damage was less extensive that patient 1 **(D)**. Four regions (black boxes; i–iv) highlight areas of limited damage (joint middle) to severe damage (outer edge). Representative staining for phosphorylated PERK **(E)** and PKR **(F)** from these four regions are shown revealing more staining in the cartilage as the damage progresses outwards but not the extensive activity in the bone as observed for patient 1. Scale bars = 100 μm.

Sections from two human MTPs, with moderate to extensive OA degeneration (33), were examined for evidence of active stress signaling and representative images shown (Figure 6; Figure S6 in Supplementary Material). Toluidine blue stained sections show that both patients had more degeneration toward the outer edge of the plateau (Figures 6A,D). Patient 1 had extensive cartilage loss down to the subchondral bone and severe damage in what remained of the cartilage (Figure 6A). Patient 2 showed signs of proteoglycan loss, cartilage fibrillation, and a small region of significant cartilage fibrillation (Figure 6D). Immunohistochemistry revealed that active PERK and PKR were present in a range of joint tissues regardless of degenerative state (described in Figure S6 in Supplementary Material). However, in patient 1 (Figure 6A), more extensive staining was observed in the cartilage that was more severely damaged and, in particular, in the bone in regions underlying significant cartilage loss (Figures 6B,C). In patient 2 (Figure 6B), the staining for active PERK and PKR was more apparent in the cartilage as the damage increased in severity toward the outer edge but the extensive bone changes were not observed (Figures 6E,F). Staining for P58^IPK^ was very limited throughout with very low or negative staining in osteocytes, bone lining cells, and chondrocytes (Figure S6 in Supplementary Material). However, positive staining was observed in the bone marrow (Figure S6O in Supplementary Material).

## Discussion

The aims of this observational study were to describe a phenotypic characterization of donated knee joints from a P58^IPK^ knockout mouse colony. This study demonstrates for the first time that knockout of P58^IPK^, the cellular inhibitor of PKR and PERK, alters bone size and volume, and leads to a degenerative joint phenotype. Epiphyseal height was significantly reduced in the femora and tibiae of null mice, which also displayed significantly narrower tibiae than those of wild-type mice. These effects may be due to the reduced body fat and reduced body weight phenotype of P58^IPK^-null mice (25) (Figure S3 in Supplementary Material), either reducing load on the bones or reflecting direct effects of hypoinsulinemia and diabetes (25, 26) on bone growth.

These data also show that P58^IPK^ plays a critical role in maintaining joint integrity. It has been well documented that C57Bl/6 mice develop spontaneous OA-like lesions as they age (38–41) and indeed this was observed in the wild-type mice used within this study. However, loss of P58^IPK^ caused significantly increased joint degeneration scores that were greater than those which occur spontaneously in the aging wild-type C57Bl/6 mice. Furthermore, joint degeneration in P58^IPK^-null mice localized to the medial femoral compartment whereas C57Bl/6 mice develop spontaneous OA-like lesions predominantly in the lateral side of the tibia-femoral joint as they age (39, 40). The lateral compartment of P58^IPK^-null mice appeared relatively protected from age-related joint damage observed in wild-type mice, which may reflect a shift of loading from lateral to medial compartments in null mice. Such altered joint biomechanics is consistent with the increased subchondral bone OARSI scores in 12–13-month-old null mice, which were much greater in the medial and less in the lateral compartments, when compared to wild-types. A major risk factor for OA is abnormal loading and factors such as increased loading through the medial side of the joint (42) and altered contact surface areas on femoral condyles versus tibial plateau, mean that load-induced changes vary across different regions of the joint (43). There is some evidence that specific mechanical loading regimes can activate PKR in articular cartilage (14), although this has not been tested in bone.

High-resolution μCT analysis revealed significant bone loss from the region beneath the subchondral plate in the MTP and across the entire femoral condyle of mice lacking P58^IPK^. This may reflect osteoblast insufficiency, enhanced resorption (44) or a combination of these, associated with the diabetic phenotype of these null mice (45, 46). Both PERK and PKR are implicated in bone cell signaling. PERK plays a crucial role in bone development, osteoblast differentiation and maturation (47) whereas inactivation of PKR reduces osteoblast differentiation and calcification (48–50) and osteoclast differentiation (51). Thus, the changes in the subchondral bone and loss of bone below the subchondral plate in P58^IPK^-null mice may reflect site-specific effects of over-activated PERK and/or PKR on bone remodeling.

A severe phenotype, characterized by complete loss of the articular cartilage from the medial tibial and femoral compartments, subchondral bone remodeling, osteophyte formation and calcification of the collateral ligaments, was observed in a third of P58^IPK^-null mice. The reason for this variability is unknown but may reflect individual variations in immune status, ER stress, or joint loading. For example, virally infected P58^IPK^-null mice show enhanced inflammatory responses (26), and cells with a high-secretory burden from P58^IPK^-null mice have reduced capacity to cope with ER stress (31). In addition, ossification of collateral ligaments, as observed in all P58^IPK^-null mice with severely damaged joints, may cause, or be a consequence of altered joint loading. Such ossification occurs in the Dunkin–Hartley guinea pig (52) and STR/ort mouse models of spontaneous knee OA (53, 54), where ossification of the collateral and patellar ligaments and Achilles tendon is thought to alter the mechanical stability of the knee, contributing to cartilage breakdown and OA (55, 56). Transection of the anterior cruciate ligament (ACL) or non-invasive ACL rupture in mice also alters joint biomechanics and results in a remarkably similar severe phenotype to that observed in P58^IPK^-null mice with ectopic bone formation within the joint capsule, significant cartilage erosion and bone sclerosis (57–59). Of note, bone and proteoglycan deposition in the collateral ligaments, significant cartilage erosion on the femur and tibia and sclerosis in the underlying bone have also been observed in aging IL-6 knockout (60) and GM3 synthase knockout mice (61). Interestingly, absence of GM3 synathase prevents the conversion of GM3 to gangliosides, which are required to suppress MMP-13 and ADMATS-5 production. Whether PKR and/or PERK signaling pathways play a role in these models remains to be determined.

Although calcification of the periarticular structures only occurred in P58^IPK^-null mice with the severe phenotype, knee degeneration occurred in all P58^IPK^-null mice, suggesting that calcification is not the initiating factor in these mice. There is no doubt, however, that the calcified/ossified tissue will change the stiffness of the periarticular structures, alter joint biomechanics and accelerate degeneration. Calcification of the Achilles tendon increased with age in wild-type mice as expected (62), but interestingly, older P58^IPK^-null mice had significantly less calcification than age-matched wild-types. This may reflect the reduced body weight of these animals or abnormal biomechanics in the knee altering loading through the Achilles.

Loss of P58^IPK^ increases levels of phosphorylated PERK and PKR (24). PERK and ER stress have been implicated in the pathogenesis of OA (5–7) and activation of PKR causes cartilage breakdown (3, 4, 63), is linked to load-induced OA (12) and is mechano-responsive (14). Consistent with this, and to our knowledge, for the first time, we demonstrate that active PKR and PERK are both expressed at the same time in a range of joint cells in human osteoarthritic tissue. Most notable is that the levels of these kinases appear to correlate with the degree of cartilage damage and that the highest expression is observed within the bone in areas underlying severe cartilage loss. In addition, there appears to be some evidence for region-specific activation of PKR and PERK in human OA, with PERK in surface zone cartilage cells and PKR in the mid zone. These observations are consistent with the notion that over-activation of the PKR pathway in P58^IPK^-null mice combined with increased loading of the medial compartment, may contribute to joint degeneration.

Given the functional cross-talk between PERK and PKR (64) and without the control of P58^IPK^, the extent of activation of these pathways may be exaggerated in these knockout mice. Stress, viral infection, and/or altered joint biomechanics in individual mice may increase inflammatory responses, heighten ER stress, and induce load related responses to cause the severe degenerative joint phenotype as seen in a cohort of these mice. In future studies, it would be advantageous to “challenge” younger P58^IPK^-null animals with an inflammatory or mechanical insult prior to the onset of overt degeneration to determine whether this initiates the severe degenerative joint phenotype. However, the only material currently available for skeletal analysis of P58^IPK^ knockout mice is that from the hind limbs we describe in this paper. To our knowledge, there are no living colonies of this knockout animal and no frozen embryos available worldwide. Given the limited material available, our study does possess some limitations, namely, the lack of correlation of morphological changes directly with over-activation of the PKR/PERK pathway, the inability to determine the extent of ER stress, the effect of the loss of function of P58^IPK^ on its role as a co-chaperone, and the relatively small sample size in each age group. That said this does not detract from the strong degenerative joint phenotype we have observed in these null animals providing the first *in vivo* evidence showing a critical role for P58^IPK^ in OA-like joint degeneration. The major changes observed in P58^IPK^ knockout bone is of particular interest in defining an important role for these pathways in degenerative joint disease.

## Author Contributions

Conception and design: Sophie J. Gilbert, Victor C. Duance, and Deborah J. Mason. Collection and assembly of data: Sophie J. Gilbert, Lee B. Meakin, Mari A. Nowell, Cleo Selina Bonnet, Warren C. Ladiges, and John Morton. Analysis and interpretation of data: Sophie J. Gilbert, Lee B. Meakin, Mari A. Nowell, Cleo Selina Bonnet, and Deborah J. Mason. Drafting of the manuscript: Sophie J. Gilbert and Deborah J. Mason. Critical revision: Sophie J. Gilbert, Lee B. Meakin, Mari A. Nowell, Cleo Selina Bonnet, Victor C. Duance, and Deborah J. Mason. Final approval of the article: Sophie J. Gilbert, Lee B. Meakin, Mari A. Nowell, Cleo Selina Bonnet, Warren C. Ladiges, John Morton, Victor C. Duance, and Deborah J. Mason.

## Conflict of Interest Statement

The authors declare that the research was conducted in the absence of any commercial or financial relationships that could be construed as a potential conflict of interest.

## Supplementary Material

The Supplementary Material for this article can be found online at http://www.frontiersin.org/Journal/10.3389/fendo.2014.00174/abstract

## Abbreviations

μCT, micro-computed tomography; BV, bone volume; BV/TV, bone volume fraction; eIF2α, eukaryotic initiation factor 2 alpha; ER, endoplasmic reticulum; GLM ANOVA, general linear model analysis of variance; IFN, interferon; LFC, lateral femoral condyle; LTP, lateral tibial plateau; MFC, medial femoral condyle; MTP, medial tibial plateau; MV, marrow volume; OA, osteoarthritis; OARSI, Osteoarthritis Research Society International; P58^IPK^, 58 kDa inhibitor protein kinase; PERK, protein kinase-like endoplasmic reticulum kinase; PKR, protein kinase R; RA, rheumatoid arthritis; TNF-α, tumor necrosis factor-alpha; TV, total tissue volume.
