# Supplementary material for: Deletion of P58IPK, the Cellular Inhibitor of the Protein Kinases PKR and PERK, Causes Bone Changes and Joint Degeneration in Mice
Source: Front Endocrinol (Lausanne). 2014 Oct 17;5:174. doi: 10.3389/fendo.2014.00174 (PMC4201149; doi:10.3389/fendo.2014.00174)
Supplement: Supplementary file 1 [file Table_1.PDF]

Suppl. Table 1. Semi-quantitative scoring system (adapted from Glasson et al[21])

Grade	Parameter 1. Osteoarthritic damage
0	Normal
0.5	Loss of Toluidine blue
1	Small fibrillations without loss of cartilage
2	Vertical clefts down to the layer immediately below the superficial layer and some loss of surface lamina
3	Vertical clefts/erosion to the calcified cartilage extending to <25% of the articular surface
4	Vertical clefts/erosion to the calcified cartilage extending to 25-50% of the articular surface
5	Vertical clefts/erosion to the calcified cartilage extending to 50-75% of the articular surface
6	Vertical clefts/erosion to the calcified cartilage extending to >75% of the articular surface
**Grade**	**Parameter 2: Subchondral bone changes**
0	Normal
1	Mild
2	Moderate
3	Severe
**Grade**	**Parameter 3: Loss of proteoglycan**
0	Normal staining of non-calcified cartilage
1	Decreased but not complete loss of Toluidine blue staining over 1-100% of the articular surface
2	Complete loss of Toluidine blue staining in the non-calcified cartilage extending to <25% of the articular surface
3	Complete loss of Toluidine blue staining in the non-calcified cartilage extending to 25-50% of the articular surface
4	Complete loss of Toluidine blue staining in the non-calcified cartilage extending to 50-75% of the articular surface
5	Complete loss of Toluidine blue staining in the non-calcified cartilage extending to >75% of the articular surface
