# Supplementary figures and images for: Deletion of P58IPK, the Cellular Inhibitor of the Protein Kinases PKR and PERK, Causes Bone Changes and Joint Degeneration in Mice
Source: Front Endocrinol (Lausanne). 2014 Oct 17;5:174. doi: 10.3389/fendo.2014.00174 (PMC4201149; doi:10.3389/fendo.2014.00174)

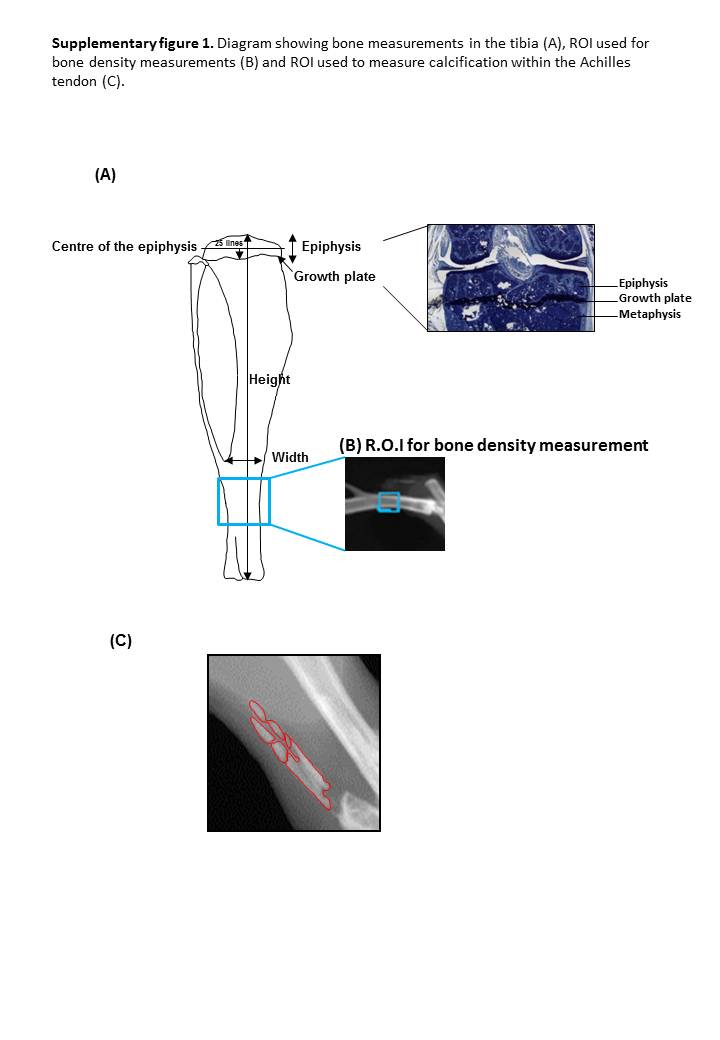

Supplement: Supplementary file 2 [file Image_1.JPEG]

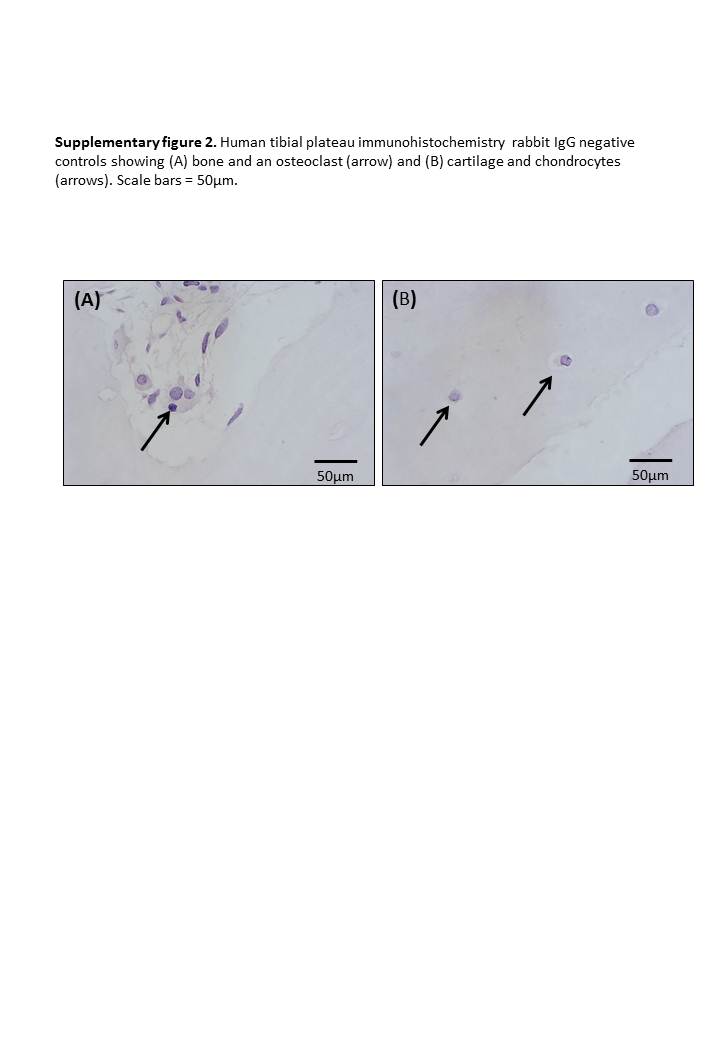

Supplement: Supplementary file 3 [file Image_2.JPEG]

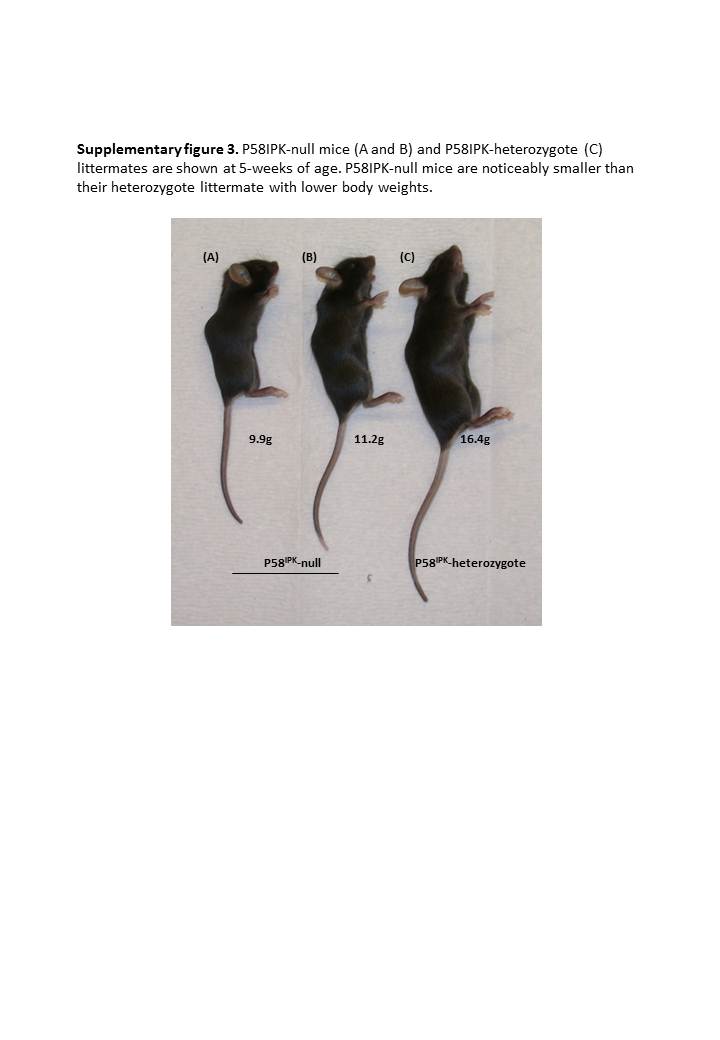

Supplement: Supplementary file 4 [file Image_3.JPEG]

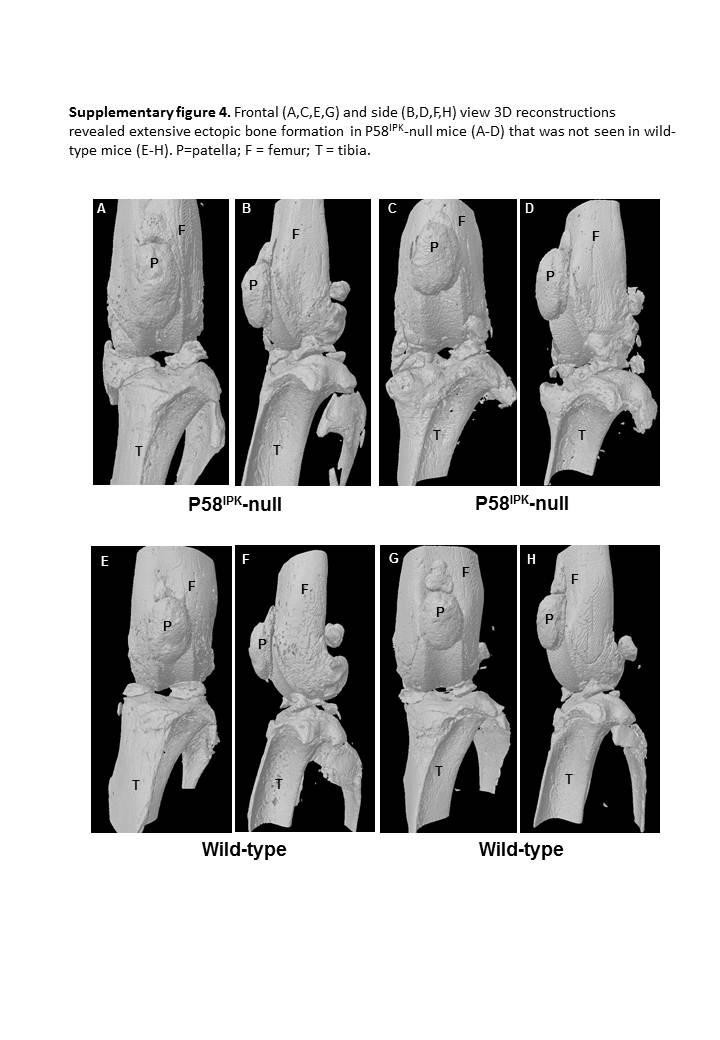

Supplement: Supplementary file 5 [file Image_4.JPEG]

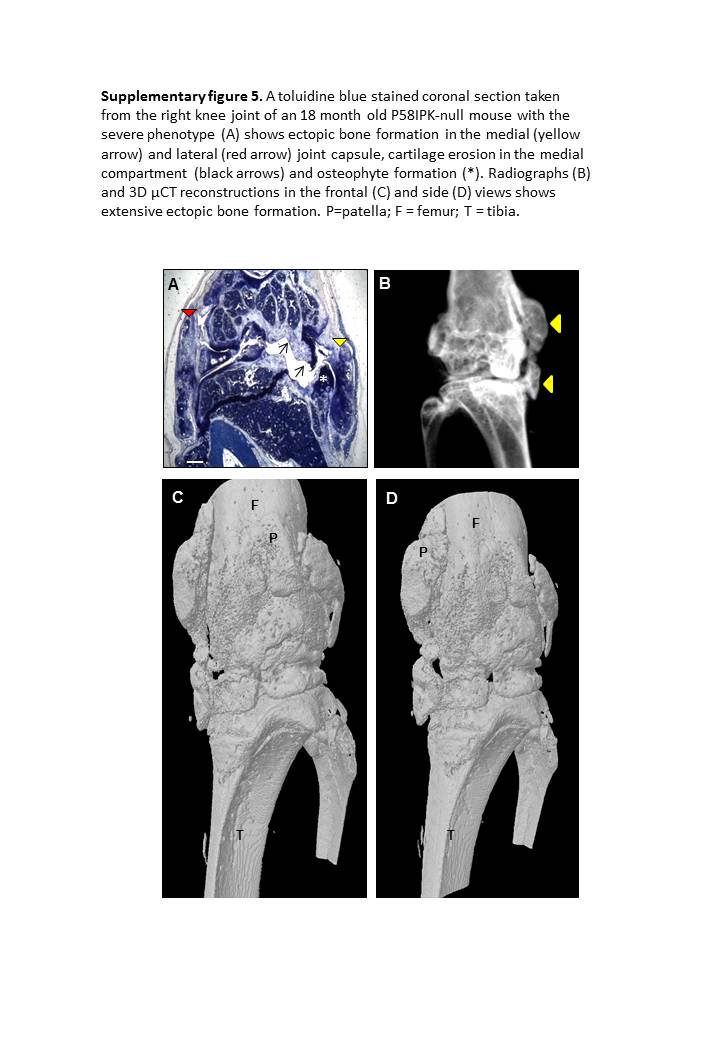

Supplement: Supplementary file 6 [file Image_5.JPEG]

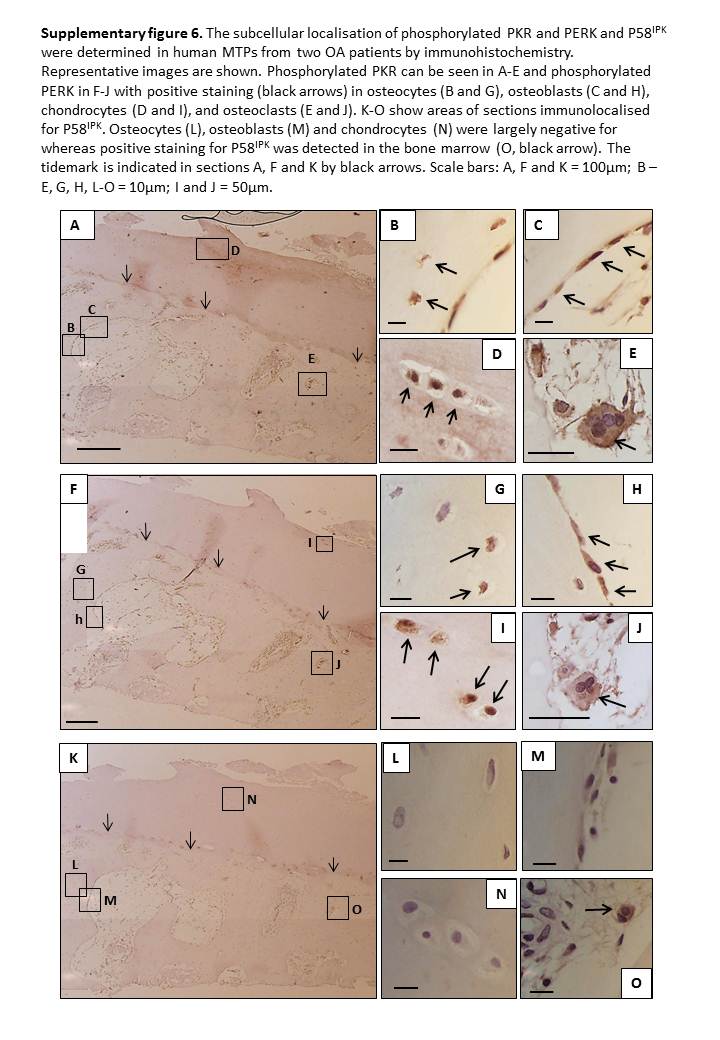

Supplement: Supplementary file 7 [file Image_6.JPEG]
